# Vitamin D status among the elderly Chinese population: a cross-sectional analysis of the 2010–2013 China national nutrition and health survey (CNNHS)

**DOI:** 10.1186/s12937-016-0224-3

**Published:** 2017-01-14

**Authors:** Jing Chen, Chunfeng Yun, Yuna He, Jianhua Piao, Lichen Yang, Xiaoguang Yang

**Affiliations:** 1 Department of Trace Element Nutrition, National Institute of Nutrition and Health, Chinese Center for Disease Control and Prevention, Room 103, Nanwei Road No. 29, Xicheng District, Beijing, 100050 People’s Republic of China; 2 Institute of Population Research, Peking University, Beijing, 100871 People’s Republic of China; 3 Department of Nutrition Surveillance, National Institute of Nutrition and Health, Chinese Center for Disease Control and Prevention, Beijing, 100050 People’s Republic of China

**Keywords:** Vitamin D, Elderly population, China, Risk factors, Environment

## Abstract

**Background:**

Vitamin D inadequacy is common among the elderly, especially within the Asian population. The vitamin D status among healthy adults in the elderly Chinese population was evaluated.

**Methods:**

A total of 6014 healthy adults aged 60 years or older (2948 men, 3066 women) participated in this descriptive cross-sectional analysis. Possible predictors of vitamin D inadequacy were evaluated via multiple logistic regression analyses.

**Results:**

The median serum 25-hydroxyvitamin D (25(OH)D) levels were 61.0 nmol/l (interquartile range (IQR) 44.3–80.6, range 5.1–154.5) for men and 53.7 nmol/l (IQR 38.8–71.0, range 6.0–190.0) for women, with 34.1% (95% confidence interval (CI) 32.4–35.8) of men and 44.0% (95% CI 42.2–45.8) of women presenting vitamin D inadequacy (25(OH)D <50 nmol/l). According to the multivariate logistic regression analyses, vitamin D inadequacy was positively correlated with female gender (*P* <0.0001), underweight (*P* = 0.0259), the spring season (*P* <0.0001), low ambient UVB levels (*P* <0.0001) and living in large cities (*P* = 0.0026). For men, vitamin D inadequacy was positively correlated with the spring season (*P* = 0.0015), low ambient UVB levels (*P* <0.0001) and living in large cities (*P* = 0.0022); for women, vitamin D inadequacy was positively correlated with the spring season (*P* = 0.0005) and low ambient UVB levels (*P* <.0001).

**Conclusions:**

Vitamin D inadequacy is prevalent among the elderly population in China. Because residing in regions with low ambient UVB levels increases the risk of vitamin D inadequacy both for men and women, vitamin D supplementation and sensible sun exposure should be encouraged, especially during the cooler seasons. Further studies are required to determine the optimal vitamin D intake and sun exposure levels to maintain sufficient vitamin D levels in the elderly Chinese population.

## Background

Vitamin D status is related to many types of health outcomes among the elderly population and can contribute to musculoskeletal complications (including proximal myopathy, bone and muscle pain, secondary hyperparathyroidism, osteoporosis, and osteomalacia) [1], chronic diseases (cancer and cardiovascular diseases) [2], autoimmune diseases (systemic lupus erythematosus, multiple sclerosis, scleroderma or systemic sclerosis, autoimmune thyroid diseases, rheumatoid arthritis and primary biliary cirrhosis) [3, 4], and psychotic disorders (depression, self-neglect and cognitive impairment) [5–7]. All these diseases can cause disability among the elderly. Compared to individuals with sufficient vitamin D levels, older women with vitamin D deficiency were significantly older, heavier, and less physically active; furthermore, these women presented with more comorbidities [8]. A healthy vitamin D status in an elderly individual is important to avoid disability. Because the percentage of people in China aged 60 years and older is expected to reach 36.5% by 2050 and 39.6% by 2100 [9], the health of the elderly Chinese population is receiving increasing levels of attention.

The primary source of vitamin D for most human populations is sunlight exposure between approximately 9:00 and 15:00 h (local solar time) during the spring, summer and autumn seasons [10]. At more extreme latitudes, the solar elevation angles and ambient UVB levels are always low; thus, the UVB exposure periods required to produce an ideal quantity of vitamin D are sometimes impractical, especially during the cooler seasons. Vitamin D requirements are thought to vary with age, yet all age groups have been shown to exhibit a high prevalence of 25-hydroxyvitamin D (25(OH)D) insufficiency and secondary hyperparathyroidism [11]. The elderly population requires more vitamin D to produce the increased 25(OH)D concentrations necessary to overcome the hyperparathyroidism associated with their diminishing renal function [11]. Currently, vitamin D deficiency among the elderly population constitutes a widespread and urgent public health issue that must be remedied.

In the present study, the prevalence of vitamin D deficiency and insufficiency in a nationally representative sample of the Chinese population over 60 years old was evaluated. The primary risk factors of inadequate vitamin D levels among the elderly Chinese population was predicted based on age, gender, BMI, ambient UVB levels in the residential regions, vitamin D supplementation levels and hypertension. National vitamin D data are instrumental to the development of appropriate health monitoring of the elderly Chinese population, and the Chinese Center for Disease Control and Prevention could use these data to identify at-risk groups and provide advice regarding the use of vitamin D supplements.

## Methods

### Study design

Data for the present analysis were extracted from the China National Nutrition and Health Survey (CNNHS), 2010–2013. This survey was a nationally representative cross-sectional study conducted by the Chinese Center for Disease Control and Prevention to assess the health and nutrition of Chinese civilians. The survey randomly selected 150 districts (urban) or counties (rural) within all thirty-one provinces, autonomous regions and municipalities directly under the central Chinese government (except Taiwan, Hong Kong and Macao). The country was divided into four strata according to socioeconomic characteristics: large cities, small to medium cities, general rural areas and poor rural areas.

### Participants and setting

This study used a stratified multistage probability sampling design and aimed to randomly survey approximately 40 people (20 men, 20 women) aged 60 years or older in each district or county. Participants who did not have adequate serum samples for 25(OH)D measurement and individuals with a history of either kidney disease or chronic liver disease were excluded. Finally, 2948 men and 3066 women were enrolled in the study. Serum samples were collected from the participants between June 2013 and March 2014, and the body mass index (BMI) was calculated as the weight (in kg) divided by the height (in m) squared. According to the Chinese definitions, underweight, overweight and obesity were defined using the BMI cut-off points of 18.5, 24 and 28 kg/m^2^, respectively [12].

### Data collection

Demographic data (age, ethnicity) and data on vitamin D supplement use and educational level were collected based on self-reports. After 5 min of rest in a seated position, blood pressure levels were measured twice from the right arm of each participant; the mean of the two measurements was used for analysis. Hypertension was defined as two separate blood pressure measurements >140/90 mmHg at least 6 h apart [13]. On the day after the participants provided written informed consent, blood samples were collected in the morning after a fasting period of 10–12 h and centrifuged within 0.5–1 h following collection. Serum samples were then aliquoted and stored at —80 °C until further analysis.

### Measurement of serum 25-hydroxyvitamin D

Serum 25(OH)D levels were quantified by using 25-Hydroxyvitamin D radioimmunoassay kits (DiaSorin-RIA; DiaSorin Inc., Stillwater, MN) on a GC-2016 gamma counter (ANHUI UCTC ZONKIA SCIENTIFIC INSTRUMENTS CO. LTD, Anhui, China). The interassay coefficients of variation (CVs) were 3.8 and 3.5% at 24.8 and 57.5 nmol/l, respectively. The DiaSorin-RIA method used in this study was in acceptable agreement with an LC-MS/MS method that has been accepted by the College of American Pathologists (CAP) as a proficient test to assess vitamin D deficiency and insufficiency [14]. In our study, participants with a serum 25(OH)D concentration lower than 30 nmol/l were stratified as vitamin D deficient; those with a concentration between 30 and 50 nmol/l as vitamin D insufficient; and those with a concentration above 50 nmol/l as vitamin D sufficient [15]. Both vitamin D deficiency and vitamin D insufficiency were grouped together as vitamin D inadequacy (25(OH)D less than 50 nmol/l).

### Ambient UVB measurements

The ambient UV radiation exposure levels for each participant was estimated using the Chinese administrative division codes of each participant’s current residential address. Latitudinal and longitudinal coordinates were obtained for each code and matched against available UVB data available from a 1° latitude × 1° longitude grid of readings of a total ozone mapping spectrometer (TOMS) mounted on the Nimbus-7 satellite; the UVB data were obtained from an archived database at the NASA Goddard Space Flight Center. This database was used to estimate the average erythemally weighted UVB dose (J/m^2^) reaching the Earth at each location between June 2013 and March 2014. The HDFtool in MATLAB 7.11.0 (R2010b) was utilized to extract relevant information from the database, and the annual average ambient UVB levels for the entire cohort were classified into tertiles.

### Statistical analysis

The participants were divided into sub-classes according to a number of hypothesized predictors for vitamin D status: regional stratum, ambient UVB level at the place of residence, season (spring, March to May; summer, June to August; autumn, September to November; and winter, December to February), age, BMI, vitamin D supplement usage, and hypertension. The Kolmogorov-Smirnov test was used to analyse the normality of each sub-class, and the results indicated that most sub-classes did not follow a normal distribution. Therefore, a Kruskal-Wallis test followed by a Mann-Whitney *U* test was conducted to examine the relationships between vitamin D levels and the hypothesized predictors. To investigate the relationship between vitamin D inadequacy and several possible predictors (e.g., age, BMI, ambient UVB level at area of residence, vitamin D supplement use, hypertension), a multinomial logistic regression analysis was performed. The statistical software package SAS version 9.2 was used for data analysis. The primary analysis was descriptive and includes the 95% confidence interval (CI). Using two-sided tests, the significance level was set at P <0.05.

## Results

As a part of the CNNHS in 2010–2013, the serum 25(OH)D levels of 6014 healthy individuals over 60 years old (man 2948, women 3066) were analysed. The median ages were 67.5 years (interquartile range (IQR) 63.2–73.2, range 60.0–100.2) for men and 66.8 years (IQR 63.0–72.5, range 60.0–98.2) for women. Among elderly men, the median BMI level was 23.2 kg/m^2^ (IQR 20.3–25.8, range 12.3–44.8) with 14.0% of the participants underweight (BMI <18.5 kg/m^2^), 29.5% overweight (BMI = 24–28 kg/m^2^) and 12.3% obese (BMI> 28 kg/m^2^). Among elderly women, the median BMI level was 23.3 kg/m^2^ (IQR 20.5–25.9, range 11.4–38.9) with 13.5% of the participants underweight (BMI <18.5 kg/m^2^), 32.5% overweight (BMI = 24–28 kg/m^2^) and 11.8% obese (BMI> 28 kg/m^2^). In addition, 29.4% of the elderly men and 29.9% of the elderly women were classified as hypertensive. In total, only 0.3% of the elderly men and 0.3% of the elderly women reported using vitamin D supplements. The baseline characteristics and 25(OH)D values of all the participants are presented in Table 1.

**Table 1:** Demographic characteristics of the elderly Chinese study participants

	Men							Women						
	n	25(OH)D (nmol/l)		Percentage with 25(OH)D levels			*P* value	n	25(OH)D (nmol/l)		Percentage with 25(OH)D levels			*P* value
		mean	SD	<30 nmol/l	30–50 nmol/l	>50 nmol/l			mean	SD	<30 nmol/l	30–50 nmol/l	>50 nmol/l	
Total	2948	63.3	25.1	7.8	26.3	65.9		3066	56.1	22.9	12	32	56	
Age group							0.7021							0.2938
60–69	1818	63.9	25.1	7.5	25.5	67		1987	56.4	23.1	11.2	32.7	56.1	
70–79	951	62.5	25	8.2	27.7	64.1		878	55.4	22.4	13.7	30.2	56.2	
80+	179	61.9	24.6	8.4	27.9	63.7		201	56	22.9	11.9	33.8	54.2	
BMI (kg/m2)							0.7826							0.3829
<18.5	414	62.7	25.9	6.8	30	63.3		398	56.1	24.5	12.6	33.4	54	
18.5–24.0	1303	63.9	24.9	7.7	25	67.3		1363	56.9	23.1	11.4	31.4	57.2	
24.0–28.0	870	63.2	25	8.2	25.4	66.4		957	55.3	22.1	13	31.5	55.6	
>28.0	361	62.2	24.9	8.6	29.1	62.3		348	55	21.8	10.9	34.5	54.6	
Ethnicity							0.006							0.2212
Han nationality	2719	63.7	25	7.3	26.2	66.5		2810	56.4	23	11.8	31.6	56.6	
Hui nationality	11	71.9	24.6	9.1	0	90.9		20	53.5	17.4	5	50	45	
Other nationality	218	57.8	25	13.8	28.9	57.3		236	52.2	20.7	14.4	36	49.6	
Regional strata							<0.0001							0.0005
Large cities	701	58.2	23	9.8	30.7	59.5		724	55.3	22.1	10.1	37.6	52.3	
Small to medium cities	875	65.6	26.5	6.6	25.9	67.4		885	55.8	23.5	12.2	33.7	54.1	
General rural areas	859	65.6	25.4	7.9	21.5	70.5		924	57.2	23.6	14.2	26.8	59	
Poor rural areas	513	62.4	23.8	6.8	29	64.1		533	55.6	21.3	10.3	30.8	58.9	
Vitamin D supplementation							0.8237							0.4676
No	2940	63.3	25.1	7.8	26.3	65.9		3057	56.1	22.9	12	32	56	
Yes	8	68.4	24.3	0	25	75		9	51.9	14	11.1	33.3	55.6	
Ambient UVB levels (J/m2)							<0.0001							<0.0001
Low	953	60.1	25.2	8.1	34	57.9		964	51.7	21.6	13.8	39.6	46.6	
Medium	953	62.9	25.3	8.6	26.9	64.5		1045	55.7	23.1	13.4	31.5	55.1	
High	1042	66.6	24.3	6.8	18.8	74.4		1057	60.5	22.9	8.9	25.6	65.5	
Hypertension							0.5523							0.6414
No	2081	63.9	25	7.5	25.9	66.6		2150	55.9	22.8	12.5	31.9	55.6	
Yes	867	61.9	25.2	8.5	27.3	64.1		916	56.6	22.9	10.7	32.3	57	
Education level							0.8044							0.0779
Illiteracy	248	61.2	24.9	9.3	27	63.7		254	55	25.9	15.8	30.3	53.9	
Literate	2700	63.5	25.1	7.7	26.3	66.1		2812	56.2	22.6	11.6	32.2	56.2	
Season							0.1786							<0.0001
Spring	125	56.1	25.1	13.6	32.8	53.6		132	49.1	18.9	16.7	40.2	43.2	
Summer	382	64.1	23.9	6.3	24.9	68.8		383	59	21.8	7.3	28.2	64.5	
Autumn	1943	63.8	25.5	7.5	26.4	66.1		2042	56.6	23.3	11.5	32.2	56.3	
Winter	498	62.6	24.2	8.6	25.5	65.9		509	53.7	22.4	16.1	32.2	51.7	

Mann-Whitney *U* test was used for comparisons of different sub-groups

Among elderly men, the median serum 25(OH)D levels were 61.0 nmol/l (IQR 44.3–80.6, range 5.1–154.5), with an estimated 7.8% (95% CI 6.9–8.8) presenting vitamin D deficiency (25(OH)D <30 nmol/l) and 26.3% (95% CI 24.7–27.9) showing signs of vitamin D insufficiency (30 nmol/l ≤25(OH)D <50 nmol/l). Among elderly women, the median serum 25(OH)D levels were 53.7 nmol/l (IQR 38.8–71.0, range 6.0–190.0), with an estimated 12.0% (95% CI 10.8–13.2) presenting vitamin D deficiency (25(OH)D <30 nmol/l) and 32.0% (95% CI 30.4–33.7) showing signs of vitamin D insufficiency (30 nmol/l ≤25(OH)D <50 nmol/l). (Table 1)

In the logistic regression analysis for all the participants (Table 2), the following factors were associated with vitamin D inadequacy (OR; 95% CI): gender (women: 1.544; 1.388–1.717; *P* <0.0001 relative to men), BMI (underweight: 1.238; 1.049–1.462; *P* = 0.0259 relative to normal weight); season (spring: 2.487; 1.849–3.346, *P* <0.0001 relative to summer); ambient UVB levels in region of residence (low: 2.233; 1.952–2.553; *P* <0 · 0001 relative to high) and regional strata (large cities: 1.287: 1.109–1.494; *P* = 0.0026 relative to general rural areas). According to a logistic regression analysis of men (Table 2), vitamin D inadequacy was associated with season (spring: 2.137; 1.398–3.268; *P* = 0.0015 relative to summer) ambient UVB levels at region of residence (low: 2.119; 1.743–2.575; *P* <0 · 0001 relative to high) and regional strata (large cities: 1.466; 1.179–1.823; *P* = 0.0022 relative to general rural areas). According to a logistic regression analysis of women (Table 2), vitamin D inadequacy was associated with season (spring: 2.858; 1.887–4.327; *P* = 0.0005 relative to summer) and ambient UVB levels at region of residence (low: 2.352; 1.954–2.832; *P* <.0001 relative to high).

**Table 2:** Associations of various lifestyle factors with vitamin D inadequacy among the elderly Chinese population

	Total (*n*=6014)		Men (*n*=2948)		Women (*n*=3066)	
	OR (95% CI)	*P* value	OR (95% CI)	*P* value	OR (95% CI)	*P* value
Gender						
Men	ref					
Women	1.544 (1.388–1.717)	<0.0001				
Age group						
60–69	ref		ref		ref	
70–79	1.066 (0.948–1.199)	0.9405	1.126 (0.952–1.332)	0.6001	1.01 (0.858–1.19)	0.5367
80+	1.149 (0.923–1.432)	0.3334	1.132 (0.816–1.572)	0.6947	1.159 (0.86–1.56)	0.3447
BMI (kg/m2)						
18.5–24.0	ref		ref		ref	
<18.5	1.238 (1.049–1.462)	0.0259	1.261 (0.994–1.6)	0.0857	1.225 (0.971–1.546)	0.1284
24.0–28.0	1.005 (0.885–1.141)	0.1451	0.974 (0.806–1.177)	0.1329	1.031 (0.868–1.226)	0.5529
>28.0	1.079 (0.904–1.289)	0.9713	1.11 (0.86–1.433)	0.7736	1.044 (0.815–1.336)	0.7716
Season						
Summer	ref		ref		ref	
Spring	2.487 (1.849–3.346)	<0.0001	2.137 (1.398–3.268)	0.0015	2.858 (1.887–4.327)	0.0005
Autumn	1.462 (1.235–1.731)	0.2201	1.269 (0.996–1.618)	0.3442	1.66 (1.313–2.098)	0.4294
Winter	1.591 (1.301–1.947)	0.6809	1.245 (0.929–1.667)	0.3491	1.977 (1.496–2.614)	0.1581
Ambient UVB (J/m2)						
High	ref		ref		ref	
Low	2.233 (1.952–2.553)	<0.0001	2.119 (1.743–2.575)	<0.0001	2.352 (1.954–2.832)	<0.0001
Medium	1.646 (1.442–1.877)	0.0939	1.665 (1.37–2.025)	0.1111	1.625 (1.358–1.944)	0.4636
Education level						
Illiteracy	ref		ref		ref	
Literate	0.854 (0.704–1.036)	0.1085	0.875 (0.661–1.158)	0.3515	0.837 (0.641–1.094)	0.1928
Region strata						
General rural areas	ref		ref		ref	
Large cities	1.287 (1.109–1.494)	0.0026	1.466 (1.179–1.823)	0.0022	1.147 (0.935–1.408)	0.2101
Small to medium cities	1.136 (0.987–1.307)	0.664	1.093 (0.887–1.346)	0.2145	1.18 (0.974–1.429)	0.0716
Poor rural areas	1.052 (0.894–1.238)	0.2912	1.234 (0.974–1.565)	0.6048	0.916 (0.732–1.145)	0.0573
Hypertension						
No	ref		ref		ref	
Yes	1.012 (0.894–1.145)	0.8529	0.911 (0.76–1.093)	0.3181	1.099 (0.928–1.301)	0.2751

## Discussion

More than one-third of the elderly Chinese participants in the present study had vitamin D inadequacies (25(OH)D <50 nmol/l), even during the summer and autumn seasons. The prevalence of vitamin D deficiency peaked at approximately 40 and 50% for men and women, respectively, during the spring season. The prevalence of vitamin D levels <50 nmol/l among Chinese adults is 34.3%, which is similar to that of the Canadian population (36.8%) [16], slightly lower than that of the European population(40.4%) [17] and higher than that of the US population (24%) [18]. In this study, the median 25(OH)D level in Chinese adults was 61.0 nmol/l, which was similar with the studies in the US [18] (median 25(OH)D_3_: 63.6 nmol/l) and Canadian populations (median 25(OH)D: 63.8 nmol/l) [16].

Seasonal variations were observed in the serum 25(OH)D concentrations among the participants, with the lowest serum 25(OH)D concentrations reported during the spring months. The 2007–2010 National Health and Nutrition Examination Survey (NHANES) provided the nationally representative serum 25(OH)D concentrations in the US, and seasonal differences in vitamin D status were also observed [18]. In addition, our result was similar to a previous study, which noted that the vitamin D status was poorest during the spring among the elderly population in northern China [19].

In this study, a significant gender difference regarding the risk of vitamin D deficiency was identified. Compared to older men, older women had a 1.544-fold increased risk of vitamin D deficiency; this result is similar with a previous study focused in Jinan (Shandong province, China), which reported that the men had a better vitamin D status than women [20]. Another study in Caucasian adults indicated that plasma 25(OH)D levels were lower in women overall, especially among older subjects [21]. The different hormone levels and lifestyles may be possible contributors to different vitamin D levels between men and women. Women are more likely to use sunglasses, sunscreen or other sun protection equipment than men [22], especially among the Chinese population [23]. In addition, multivariate linear regression analysis showed that lower vitamin D levels were strongly associated with sunscreen use [24].

Elderly individuals who were underweight had a higher risk of vitamin D deficiency than those at a normal weight. There has been extensive discussion regarding the relationship between vitamin D deficiency and body weight, with many studies reporting an inverse association between vitamin D deficiency and obesity [25–28]. The long-standing concept that fat tissue is a storage site for vitamin D has led many researchers to believe that the association between vitamin D deficiency and obesity may due to increased metabolic clearance of vitamin D through enhanced uptake in fat tissue [29] and/or decreased bioavailability of vitamin D once it is deposited in fat tissue [30]. However, identify an association between increased body weight and vitamin D deficiency was not identified in the present study. Furthermore, the low percentage of participants with a high BMI might have impaired the ability to detect this association. Blum et al. [31] reported that fat tissue and serum vitamin D concentrations were positively correlated, which indicated that we need to rediscover the cause responsible for the association between vitamin D deficiency and body weight. In our study, underweight individuals were at higher risk for vitamin D deficiency, an observation that was similar to those of previous reports [21, 32]. Although underweight people are more likely to be malnourished, which could result in vitamin D deficiency [32], the real cause is still unknown.

Men living in large cities are at increased risk for vitamin D deficiency than those living in rural areas. Air pollution in the cities can decrease the ambient UVB level and affected the vitamin D health of the urban population [33]; this may be an explanation why, in our study, older people living in big cities presented a poorer vitamin D status than rural residents. However, the differences between rural and urban citizens could not be observed among older women, who were more likely to be vitamin D deficient than men in this study. Almost half of the elderly women in China were categorized with vitamin D inadequacy; therefore, the different vitamin D statuses between the urban and rural populations were not as significant among older Chinese women.

Based on our logistic regression, the elderly population (men and women) living in regions with low ambient UVB levels are at higher risk of vitamin D inadequacy than individuals living in areas with high or medium ambient UVB levels. As skin exposure to UVB rays serves is a primary source of vitamin D for most people, the exposure period required to achieve an equivalent oral dose of approximately 1000 IU vitamin D increases with increasing latitude [10]; therefore, ambient UVB levels were an expected predictor of vitamin D inadequacy in our study. However, vitamin D deficiency is also common in regions with plentiful sunlight [34, 35]. In China, there was an increasing trend in the risk of vitamin D deficiency among pregnant women exposed to low ambient UVB levels [36].

The study presents some limitations. Several studies have shown that sun exposure is an important determinant of serum 25(OH)D levels [37, 38]; however, we were unable to determine the duration and region of sun exposure for each participant. Instead, we used the ambient UVB levels to estimate the sun exposure levels for the participants and found that these ambient UVB levels affect the serum 25(OH)D levels among the elderly Chinese population. Dietary calcium intake is another crucial factor that affects vitamin D status, and some experimental studies have shown that dietary calcium deficiency can lead to secondary vitamin D deficiency [39]. Unfortunately, we did not estimate the dietary calcium intake levels for each participant.

The study was a descriptive cross-sectional analysis. These results can only inform us of associations between potential predictive factors and vitamin D deficiency levels, but we cannot assess the root causes of the increased incidence of vitamin D deficiency and insufficiency among the elderly Chinese population, which may be another limitation of our study. Although the study was cross-sectional in nature, the findings suggest that exposure to elevated UVB levels may prevent vitamin D deficiency and insufficiency.

## Conclusions

Vitamin D deficiency and insufficiency were common among the elderly Chinese population, with almost one-third of the participants classified as having vitamin D deficiency or insufficiency. These conditions are common among older women, individuals residing in regions with low ambient UVB levels and older men living in large cities. During the spring season, vitamin D deficiency and insufficiency are more pronounced than during any other season. Further studies must identify optimal durations of outdoor activity and the necessary vitamin D intake levels to maintain sufficient vitamin D levels (25(OH)D >50 nmol/l) among the elderly population in China.

## References

[1] Lips P (2001). Vitamin D, deficiency and secondary hyperparathyroidism in the elderly: consequences for bone loss and fractures and therapeutic implications. Endocr Rev.

[2] Michaelsson K, Baron JA, Snellman G, Gedeborg R, Byberg L, Sundstrom J (2010). Plasma vitamin D and mortality in older men: a community-based prospective cohort study. Am J Clin Nutr.

[3] Abaza NM, El-Mallah RM, Shaaban A, Mobasher SA, Al-Hassanein KF, Abdel Zaher AA (2016). Vitamin D deficiency in Egyptian systemic lupus erythematosus patients: how prevalent and does it impact disease activity?. Integr Med Insights.

[4] Rosen Y, Daich J, Soliman I, Brathwaite E, Shoenfeld Y (2016). Vitamin D and autoimmunity. Scand J Rheumatol.

[5] Aung K, Burnett J, Smith SM, Dyer CB (2006). Vitamin D deficiency associated with self-neglect in the elderly. J Elder Abuse Negl.

[6] Barnard K, Colon-Emeric C (2010). Extraskeletal effects of vitamin D in older adults: cardiovascular disease, mortality, mood, and cognition. Am J Geriatr Pharmacother.

[7] Chei CL, Raman P, Yin ZX, Shi XM, Zeng Y, Matchar DB (2014). Vitamin D levels and cognition in elderly adults in China. J Am Geriatr Soc.

[8] Bolland MJ, Bacon CJ, Horne AM, Mason BH, Ames RW, Wang TK (2010). Vitamin D insufficiency and health outcomes over 5 y in older women. Am J Clin Nutr.

[9] United Nations Department of Economic and Social Affairs, Population Division (2015). World population prospects: the 2015 revision, key findings and advance tables. Working paper No. ESA/P/WP.241.

[10] Webb AR, Engelsen O (2006). Calculated ultraviolet exposure levels for a healthy vitamin D status. Photochem Photobiol.

[11] Vieth R, Ladak Y, Walfish PG (2003). Age-related changes in the 25-hydroxyvitamin D versus parathyroid hormone relationship suggest a different reason why older adults require more vitamin D. J Clin Endocrinol Metab.

[12] National Health and Family Planning Commission of the People’s Republic of China (2013). People’s republic of China health industry standards: criteria of weight for adults.

[13] National High Blood Pressure Education Program Working Group (1994). National high blood pressure education program working group report on hypertension in the elderly. Hypertension.

[14] Yun C, Chen J, Yang C, Li Y, Piao J, Yang X (2015). Comparison of two 25-hydroxyvitamin D immunoassays to liquid chromatography-tandem mass spectrometry in assessing samples from the Chinese population. Clin Chim Acta.

[15] Francis R, Aspray T, Fraser W, Gittoes N, Javaid K, MacDonald H. Vitamin D and bone health: a practical clinical guideline for patient management. Scotland: National Osteoporosis Society; 2013.

[16] Sarafin K, Durazo-Arvizu R, Tian L, Phinney KW, Tai S, Camara JE (2015). Standardizing 25-hydroxyvitamin D values from the Canadian health measures survey. Am J Clin Nutr.

[17] Cashman KD, Dowling KG, Skrabakova Z, Gonzalez-Gross M, Valtuena J, De Henauw S (2016). Vitamin D deficiency in Europe: pandemic?. Am J Clin Nutr.

[18] Schleicher RL, Sternberg MR, Looker AC, Yetley EA, Lacher DA, Sempos CT (2016). national estimates of serum total 25-hydroxyvitamin D and metabolite concentrations measured by liquid chromatography-tandem mass spectrometry in the US population during 2007–2010. J Nutr.

[19] Yan L, Prentice A, Zhang H, Wang X, Stirling DM, Golden MM (2000). Vitamin D status and parathyroid hormone concentrations in Chinese women and men from north-east of the People’s Republic of China. Eur J Clin Nutr.

[20] Feng X, Guo T, Wang Y, Kang D, Che X, Zhang H (2016). The vitamin D status and its effects on life quality among the elderly in Jinan, China. Arch Gerontol Geriatr.

[21] Touvier M, Deschasaux M, Montourcy M, Sutton A, Charnaux N, Kesse-Guyot E (2015). Determinants of vitamin D status in Caucasian adults: influence of sun exposure, dietary intake, sociodemographic, lifestyle, anthropometric, and genetic factors. J Invest Dermatol.

[22] Holman DM, Berkowitz Z, Guy GP, Hawkins NA, Saraiya M, Watson M (2015). Patterns of sunscreen use on the face and other exposed skin among US adults. J Am Acad Dermatol.

[23] Cheng S, Lian S, Hao Y, Kang N, Li S, Nie Y (2010). Sun-exposure knowledge and protection behavior in a North Chinese population: a questionnaire-based study. Photodermatol Photoimmunol Photomed.

[24] Golan-Cohen A, Merzon E, Alhin O, Kitai E, Fogelman Y (2016). Blood levels of vitamin D and health-functional status in asymptomatic individuals: a cross sectional study. J Eval Clin Pract.

[25] Holick MF, Chen TC (2008). Vitamin D deficiency: a worldwide problem with health consequences. Am J Clin Nutr.

[26] Gilbert-Diamond D, Baylin A, Mora-Plazas M, Marin C, Arsenault JE, Hughes MD (2010). Vitamin D deficiency and anthropometric indicators of adiposity in school-age children: a prospective study. Am J Clin Nutr.

[27] Tran B, Armstrong BK, McGeechan K, Ebeling PR, English DR, Kimlin MG (2013). Predicting vitamin D deficiency in older Australian adults. Clin Endocrinol (Oxf).

[28] Souberbielle JC, Massart C, Brailly-Tabard S, Cavalier E, Chanson P. Prevalence and determinants of vitamin D deficiency in healthy French adults: the VARIETE study. Endocrine. 2016;1–8. https://www.ncbi.nlm.nih.gov/pubmed/?term=Prevalence+and+determinants+of+vitamin+D+deficiency+in+healthy+French+adults%3A+the+VARIETE+study. 10.1007/s12020-016-0960-3 27106800

[29] Liel Y, Ulmer E, Shary J, Hollis BW, Bell NH (1988). Low circulating vitamin D in obesity. Calcif Tissue Int.

[30] Wortsman J, Matsuoka LY, Chen TC, Lu Z, Holick MF (2000). Decreased bioavailability of vitamin D in obesity. Am J Clin Nutr.

[31] Blum M, Dolnikowski G, Seyoum E, Harris SS, Booth SL, Peterson J (2008). Vitamin D(3) in fat tissue. Endocrine.

[32] Kim KJ, Kim YJ, Kim SH, An JH, Yoo HJ, Kim HY (2015). Vitamin D status and associated metabolic risk factors among North Korean refugees in South Korea: a cross-sectional study. BMJ Open.

[33] Agarwal KS, Mughal MZ, Upadhyay P, Berry JL, Mawer EB, Puliyel JM (2002). The impact of atmospheric pollution on vitamin D status of infants and toddlers in Delhi. India Arch Dis Child.

[34] Trilok Kumar G, Chugh R, Eggersdorfer M (2015). Poor vitamin D status in healthy populations in India: a review of current evidence. Int J Vitam Nutr Res.

[35] Smith G, Wimalawansa SJ, Laillou A, Sophonneary P, Un S, Hong R (2016). High prevalence of vitamin D deficiency in Cambodian women: a common deficiency in a sunny country. Nutrients.

[36] Yun C, Chen J, He Y, Mao D, Wang R, Zhang Y, et al. Vitamin D deficiency prevalence and risk factors among pregnant Chinese women. Public Health Nutr. 2015;1–9. https://www.ncbi.nlm.nih.gov/pubmed/26585546. 10.1017/S1368980015002980 PMC10261554 26585546

[37] Brot C, Vestergaard P, Kolthoff N, Gram J, Hermann AP, Sorensen OH (2001). Vitamin D status and its adequacy in healthy Danish perimenopausal women: relationships to dietary intake, sun exposure and serum parathyroid hormone. Br J Nutr.

[38] Maeda SS, Kunii IS, Hayashi L, Lazaretti-Castro M (2007). The effect of sun exposure on 25-hydroxyvitamin D concentrations in young healthy subjects living in the city of Sao Paulo, Brazil. Braz J Med Biol Res.

[39] Clements MR, Johnson L, Fraser DR (1987). A new mechanism for induced vitamin D deficiency in calcium deprivation. Nature.

